# The landscape of transposable elements and satellite DNAs in the genome of a dioecious plant spinach (*Spinacia oleracea* L.)

**DOI:** 10.1186/s13100-019-0147-6

**Published:** 2019-01-18

**Authors:** Shu-Fen Li, Yu-Jiao Guo, Jia-Rong Li, Dong-Xu Zhang, Bing-Xiao Wang, Ning Li, Chuan-Liang Deng, Wu-Jun Gao

**Affiliations:** 1 College of Life Sciences, Henan Normal University, Xinxiang, 453007 China; 2 College of Life Science, Shanxi Datong University, Datong, 037009 China

**Keywords:** Repetitive sequence, Satellite DNA, Sex chromosome, Spinach, Transposable elements

## Abstract

**Background:**

Repetitive sequences, including transposable elements (TEs) and satellite DNAs, occupy a considerable portion of plant genomes. Analysis of the repeat fraction benefits the understanding of genome structure and evolution. Spinach (*Spinacia oleracea* L.), an important vegetable crop, is also a model dioecious plant species for studying sex determination and sex chromosome evolution. However, the repetitive sequences of the spinach genome have not been fully investigated.

**Results:**

We extensively analyzed the repetitive components of draft spinach genome, especially TEs and satellites, by different strategies. A total of 16,002 full-length TEs were identified. Among the most abundant long terminal repeat (LTR) retrotransposons (REs), *Copia* elements were overrepresented compared with *Gypsy* ones. Angela was the most dominating *Copia* lineage; Ogre/Tat was the most abundant *Gypsy* lineage. The mean insertion age of LTR-REs was 1.42 million years; approximately 83.7% of these elements were retrotransposed during the last two million years. RepeatMasker totally masked about 64.05% of the spinach genome, with LTR-REs, non-LTR-REs, and DNA transposons occupying 49.2, 2.4, and 5.6%, respectively. Fluorescence in situ hybridization (FISH) analysis showed that most LTR-REs dispersed all over the chromosomes, by contrast, elements of CRM lineage were distributed at the centromeric region of all chromosomes. In addition, Ogre/Tat lineage mainly accumulated on sex chromosomes, and satellites Spsat2 and Spsat3 were exclusively located at the telomeric region of the short arm of sex chromosomes.

**Conclusions:**

We reliably annotated the TE fraction of the draft genome of spinach. FISH analysis indicates that Ogre/Tat lineage and the sex chromosome-specific satellites DNAs might participate in sex chromosome formation and evolution. Based on FISH signals of microsatellites, together with 45S rDNA, a fine karyotype of spinach was established. This study improves our knowledge of repetitive sequence organization in spinach genome and aids in accurate spinach karyotype construction.

**Electronic supplementary material:**

The online version of this article (10.1186/s13100-019-0147-6) contains supplementary material, which is available to authorized users.

## Background

A substantial fraction of plant genomes is occupied by repetitive DNA, which mainly includes transposable elements (TEs) and satellite DNAs. TEs are DNA fragments that have the ability to move from one part of a genome to another, often accounting for a large proportion of the plant genome. They are categorized into two distinct classes based on structural feature and transposition pattern. Class I elements are also known as retrotransposons (REs), which can transpose via an RNA intermediate and self-replicate when transposed. Class II elements, also called DNA transposons, can move by direct “cut-and-paste” mode. Given that REs can increase their copy numbers after being transposed, they are usually the most abundant repetitive elements, especially long terminal repeat (LTR) REs. For example, in maize, REs and LTR-REs constitute 75.6 and 70.1% of the genome, respectively, whereas DNA transposons occupy 8.6% [1]. LTR-REs exhibit typical structural features, such as the presence of LTRs at both ends, promoter and RNA processing signals, and flanking target site duplications (TSD) [2]. Near the inner 5′ LTR boundary and the inner 3′ boundary are the primer-binding site (PBS) motif and polypurine tract (PPT), which respectively provide the signals required for the minus and plus DNA strand synthesis. The internal region of REs is generally divided into two open reading frames: GAG and POL. GAG encodes for a structural protein that packages the transcript into a virus-like particle. POL codes a polyprotein with protease, integrase (INT), reverse transcriptase (RT), and RNAseH (RH), which are essential for the replication and integration of elements in target regions [3]. Satellite DNA, also known as tandem repeat, is another type of repetitive element widely distributed in plants. It consists of a large number of repeat units (50–1000 bp) that are organized in tandem arrays [4].

These repetitive sequences are recognized to play important roles in various processes, such as in genome evolution [5, 6], chromosomal rearrangement [7], gene creation and regulation [8, 9]. In addition, TEs and satellite DNAs can participate in various processes of plant sex chromosome evolution; such processes include recombination suppression, diversification of sex chromosome structure and morphology, sex chromosome degeneration, and dosage compensation [10, 11]. Thus, identification and annotation of TEs and satellites of the genomes of dioecious plants will lay foundation for further investigation of sex chromosome evolution.

Spinach (*Spinacia oleracea* L.) is an annual or biennial dioecious herbaceous plant belonging to *Spinacia* genus of Chenopodioideae family. The spinach genome is approximately 989 Mbp, with 2n = 2x = 12 chromosomes. As a dioecious species, the sex type of spinach is determined by X and Y chromosomes. The X and Y chromosomes are homomorphic, indicating an early evolutionary stage of sex chromosomes in spinach. Current cytological research have demonstrated sex chromosomes as the longest pair of chromosomes [12, 13]. Given that repetitive sequences exhibit important effects on genome structure and evolution, a comprehensive analysis of repetitive sequences of the spinach genome is beneficial for understanding of the genome structure and evolution of spinach, especially of its sex chromosomes. A draft spinach genome has recently been published [14]. Although the authors annotated the repetitive sequence fraction of the draft genome, they only used the results of LTRharvest for TE identification and further annotation. A comparative study showed that LTRharvest without other software and methods for verification yields considerably high level of false positive ratio [15]. Thus, understanding of the repetitive sequences of spinach still needs further comprehensive analysis. Furthermore, the chromosome location, phylogenetic analysis, and evolution patterns of repetitive sequences of spinach genome remain to be studied.

Accurate identification and annotation of TE fraction in whole genome sequences are challenging tasks owing to the significant diversity of TEs [15]. Currently, a number of different methods and tools have been developed for detecting TEs in assembled genomes. Three strategies are commonly used: homology-based, signature-based, and de novo approaches [15, 16]. Signature-based tools rely on the typical structure of a particular TE type and can detect individual full-length TEs, benefitting the investigation of the TE structure, variation, and evolution [17, 18]. For comprehensive and reliable annotation of a given genome, the adoption of combined approaches with downstream verification has been shown to be the best strategy [19, 20].

In this study, based on the recently published draft spinach genome, we used different methods to identify and annotate the repetitive sequence fraction of the genome, with focus on TEs and satellite DNAs. We first used signature-based methods to identify full-length TEs and extensively analyzed the phylogeny, distribution, and insertion time of LTR-REs. Combined methods, including homology-based, signature-based, and de novo approaches, were then performed for annotating the TE fraction of the whole genome. We also analyzed the location pattern of different groups of TEs and satellite DNA using fluorescence in situ hybridization (FISH). This study can provide useful information for understanding the spinach genome structure with respect to TEs and satellites.

## Results

### Identification and annotation of full-length TEs

A total of 16,002 full-length TEs were detected in spinach draft genome using signature-based strategies. This dataset included 11,640 LTR-REs, 1020 non-LTR-REs, and 3342 DNA transposons. The full-length TEs totally comprised 125,231,331 bp, accounting for 12.57% of the draft spinach genome.

#### LTR-REs

LTR retroelements were identified using LTRharvest, and internal sequences were annotated using LTRdigest. First, LTRharvest predicted the presence of 17,734 sequences harboring two relatively intact LTRs and flanking TSDs. The 5′ and 3′ ends of both LTRs were flanked by TG and CA. After LTRdigest analyses, we identified 11,640 putative full-length LTR-REs with PPT, or PBS sites, or at least one typical protein domain (Additional file 1). For 3290 and 2173 elements, the putative PBS and PPT were identified, respectively. A total of 4048 elements showed all typical protein domains of LTR-REs, but only 137 elements showed all the putative PBS, PPT, and at least one typical protein domain. The isolated LTR-REs covered a total of 110,438,058 bp, accounting for 11.09% of the whole genome. The LTR-REs ranged from 1191 bp to 22,984 bp in size. The mean length was 9787 bp, with a standard deviation of 4625 bp. The recorded putative LTRs showed a mean length of 689 bp, with large length variability (standard deviation =549 bp).

The LTR-REs were classified into *Copia* or *Gypsy* superfamilies based on the order of the POL protein domains and on similarity searches against different public RE databases. The results showed that 5303 elements (45.6%) belonged to *Copia* superfamily, whereas 3709 REs (31.9%) were designated as *Gypsy* elements. There were still 2628 elements were classified as unknown because they lacked distinct protein-coding sequences sufficient for classification (Table 1).

**Table 1:** Identification of full-length TEs in spinach genome

Class	Order	Superfamily	No.	Total length (bp)	Percentage of genome (%)
Retrotransposons	LTR	*Copia*	5303	51,884,973	5.21
		*Gypsy*	3709	40,349,864	4.05
		Unclassifed	2628	18,203,221	1.83
	LINE	CRE	642	2,981,624	0.30
		RTE	149	824,520	0.08
		I	65	400,921	0.04
		Tad1	46	287,739	0.03
		R1	30	149,274	0.15
		Rex	25	150,849	0.02
		R2	22	90,843	0.01
		others	41	227,457	0.02
	subtotal		12,660	115,551,285	11.60
DNA transposons	TIR	Tc1-Mariner	693	175,762	0.02
		hAT	397	144,071	0.01
		CMC-EnSpm	290	83,402	0.01
		MULE-MuDR	167	49,524	0.01
		PIF	26	9245	0.00
		others	27	7899	0.00
	MITE		445	160,590	0.02
	Helitron		1297	9,049,533	0.91
	subtotal		3342	9,680,026	0.97
Total			16,002	125,231,331	12.57

**Fig. 1:**
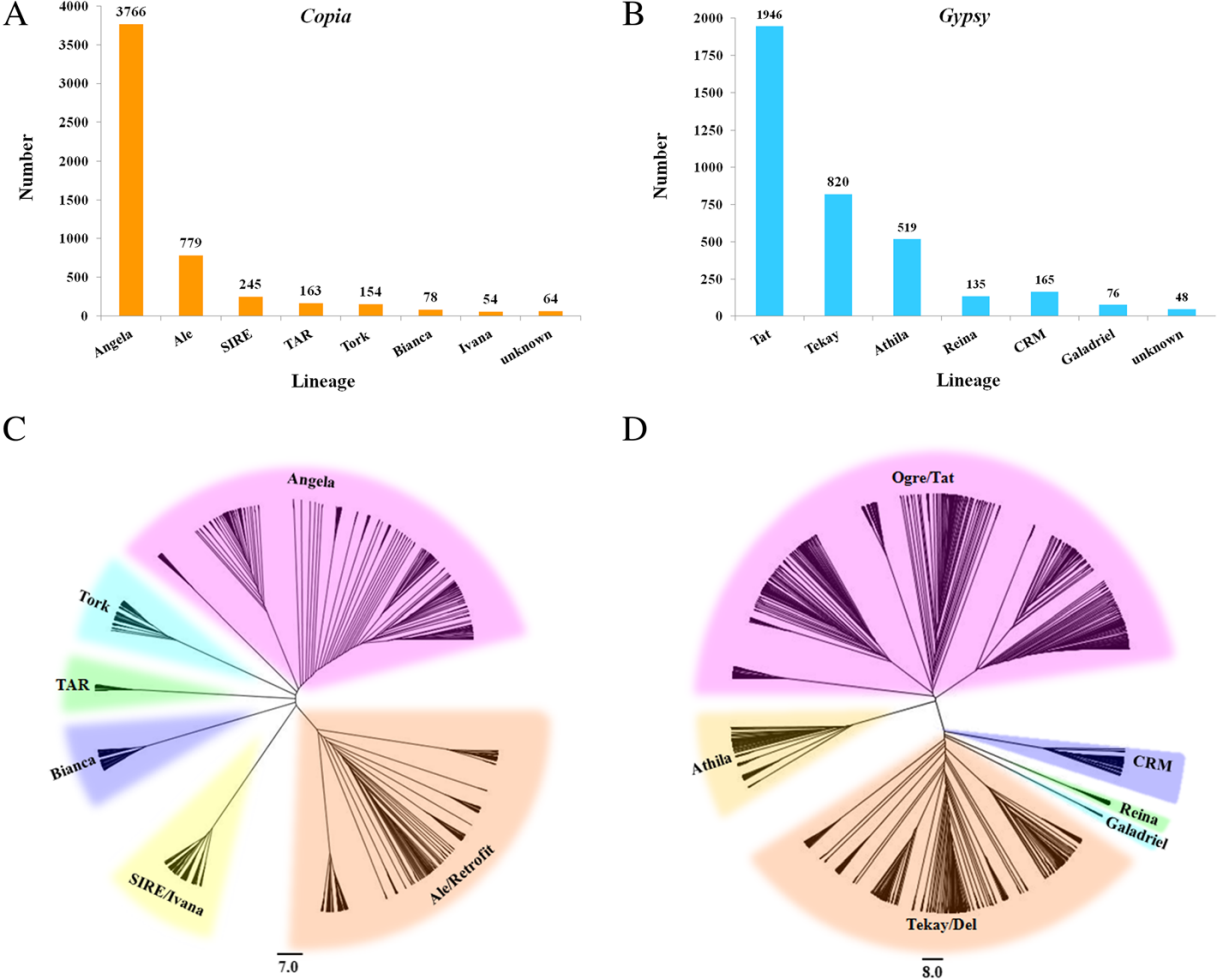
Number and phylogenetic analysis of full-length REs identified in the spinach genome and were subdivided into superfamilies and lineages. The numbers of full-length retroelements in different lineages of *Copia* (**a**) and *Gypsy* (**b**) are shown. **c** and **d** represent the phylogenetic tree of RT domain protein sequences in different lineages of *Copia* and *Gypsy*, respectively. The scale bars indicate the average number of amino acid substitutions per site

The identified full-length REs were further analyzed for the presence of five typical RE protein domains (retrotranscriptase, RH, INT, protease, and GAG). Based on the similarity to lineage-specific RE protein domains, the *Copia* elements were subdivided into seven lineages, whereas the *Gypsy* elements belonged to six lineages (Fig. 1). The remaining 64 *Copia* and 48 *Gypsy* members were defined as unknown owing to the absence of sufficient similarity to known lineage-specific RE protein domains.

Among *Copia* elements, Angela lineage was predominant, accounting for more than 70% the full-length *Copia* elements, followed by Ale/Retrofit, SIRE, TAR, and Tork. Bianca and Ivana elements were the rarest, together representing 2.5% of the *Copia* elements (Fig. 1). Analyses of *Gypsy* elements showed that Ogre/Tat, Tekay/Del, and Athila were the most common types of lineages. The other lineages, such as CRM, Reina, and Galadriel, were also identified but were less abundant, collectively accounting for 10.2% of the total *Gypsy* elements.

To determine the phylogenetic relationship of *Copia* and *Gypsy* REs, two phylogenetic trees were constructed based on the RT sequences of *Copia* and *Gypsy* REs. As shown in the evolutionary dendrograms (Fig. 1), among *Copia* REs, the Angela and Ale/Retrofit lineages showed higher variability, and could be further classified into three and two groups, respectively. SIRE and Ivana showed close relationship as they were clustered as one clade. The other lineages showed high homogeneity, as all sequences were clustered into a single clade for Tork, TAR, and Bianca (Fig. 1c). As for *Gypsy* REs, the most abundant Ogre/Tat lineage could be further classified into four groups, indicating the notable sequence diversity among this lineage. Ogre/Tat and Athila lineages both belonged to non-Chromovirus elements, and they showed close relationship and were grouped into one large clade. The other four superfamilies, Tekay/Del, Galadriel, Reina, and CRM, belonged to Chromovirus *Gypsy* REs and were clustered together (Fig. 1d).

#### Non-LTR-REs

A total of 1020 full-length non-LTR-REs were identified, occupying 5,113,227 bp and representing 0.5% of the spinach draft genome. The detected non-LTR REs all belonged to LINE order and included 12 superfamilies (Table 1). Among the different superfamilies, CRE elements were the most abundant, followed by RTE, I, and Tad1; the other superfamilies were rarely observed.

#### DNA transposons

The search for DNA transposons resulted in 1600 sequences classified as terminal inverted repeat (TIR) elements, 1297 as Helitron elements, and 445 as unknown miniature inverted repeat transposable elements (MITEs) (Table 1). Approximately 43.3% of the DNA transposons from the TIR order belonged to the superfamily Tc1-Mariner. Other identified elements belonged to hAT, CMC-EnSpm, MULE-MuDR, and PIF superfamilies. A total of 67 elements also belonged to other superfamilies, such as Dada, Maverick, and Kolobok (Table 1).

### Insertion time analysis of LTR-REs

**Fig. 2:**
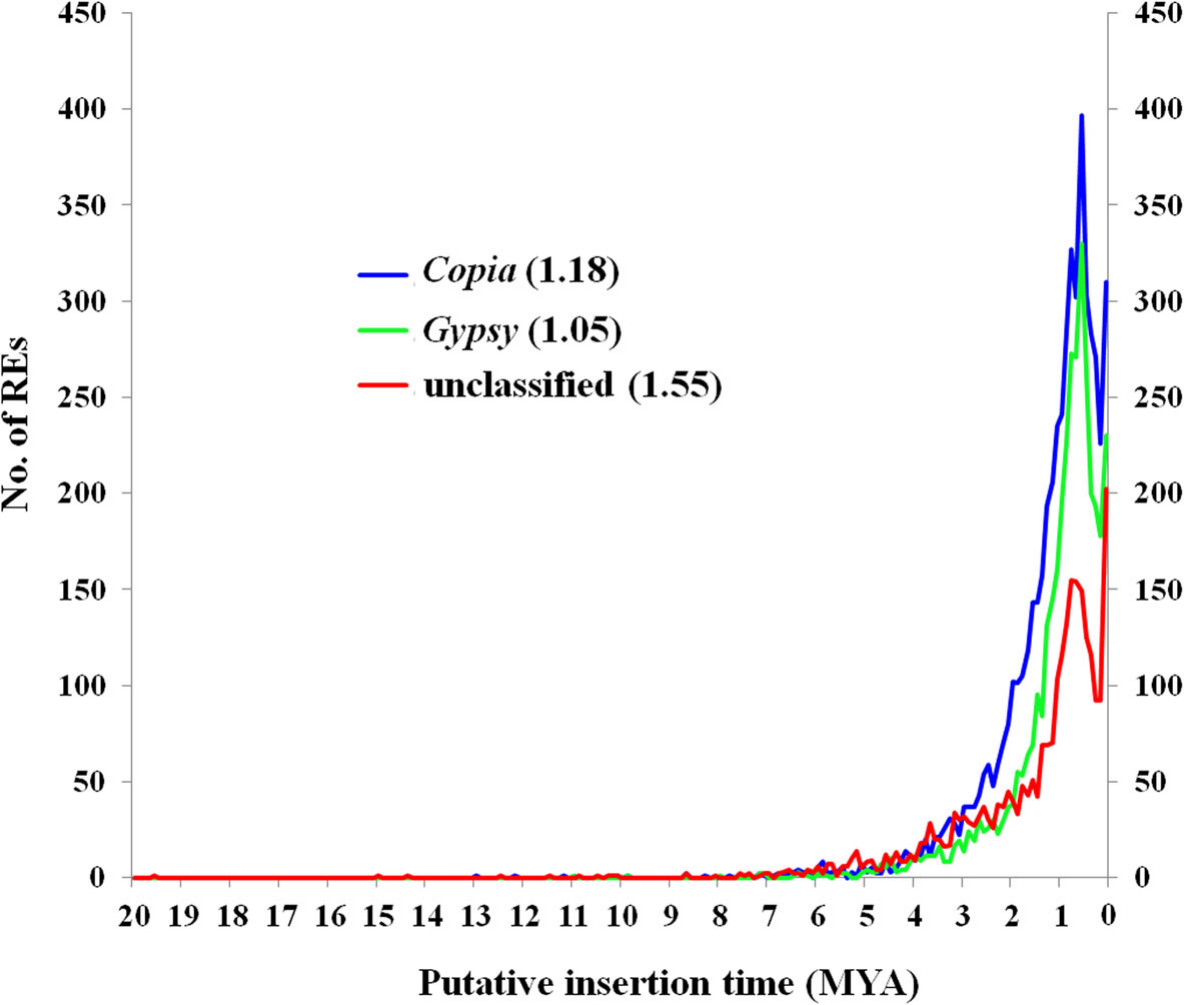
Distribution of *Copia*, *Gypsy*, and unknown full-length LTR-REs according to their estimated insertion ages (MY). The mean insertion age for each superfamily is presented

**Fig. 3:**
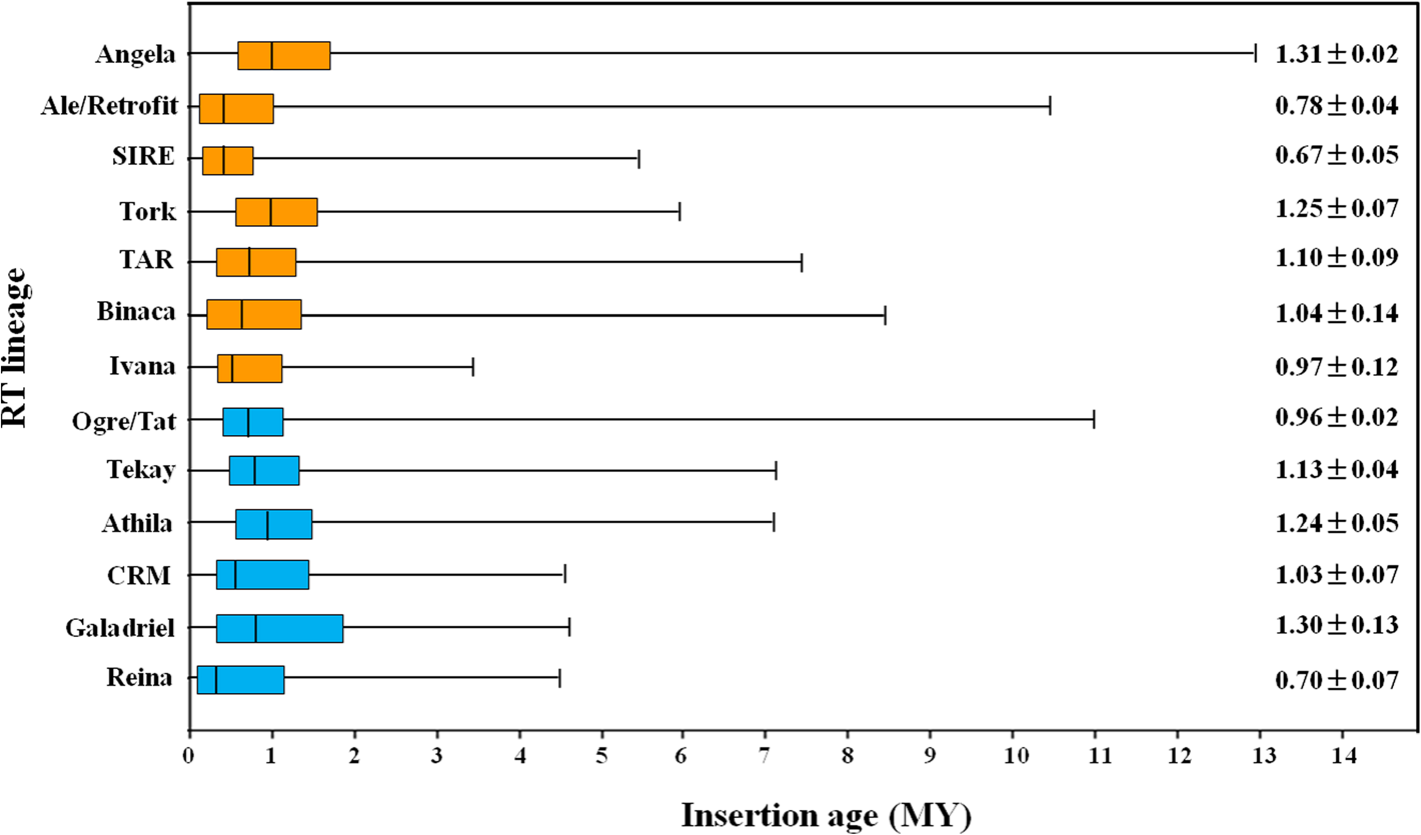
Putative insertion ages of the main spinach LTR-RE lineages. The boxes represent 25–75% of each LTR-RE lineage, whiskers represent the whole range of values, and vertical lines in the box represent the medians of distribution. For each lineage, the mean insertion age (±SE) is reported

According to nucleotide substitution between two terminal LTRs of each LTR-REs, we estimated the insertion time of the identified LTR-REs. The putative mean age of analyzed LTR-REs is 1.42 million years (MY). Nearly 83.7% of them inserted in the last 2 MY, with a peak of activity was observed at ~ 0.5–0.8 MY. In general, the *Copia* and *Gypsy* elements were younger than the unknown elements. The mean insertion ages of *Copia*, *Gypsy*, and unknown elements are 1.18, 1.05, and 1.55 MY, respectively (Fig. 2). The oldest element was an unknown element, with a putative insertion age of 19.6 MY. Among the > 10 MY elements, 4, 1, and 10 belonged to *Copia*, *Gypsy*, and unknown elements, respectively. Analysis of insertion dates of the main *Copia* and *Gypsy* lineages showed that different lineages underwent retrotransposition in different time spans (Fig. 3). The youngest lineage was Reina, which belonged to the *Gypsy* lineage, with mean and median insertion ages of 0.70 ± 0.07 and 0.35 MY, respectively. By contrast, in a *Copia* lineage, Angela showed the oldest putative insertion date, with mean and median insertion ages of 1.31 ± 0.02 and of 1.00 MY, respectively.

### Repeat composition of the spinach draft genome

**Fig. 4:**
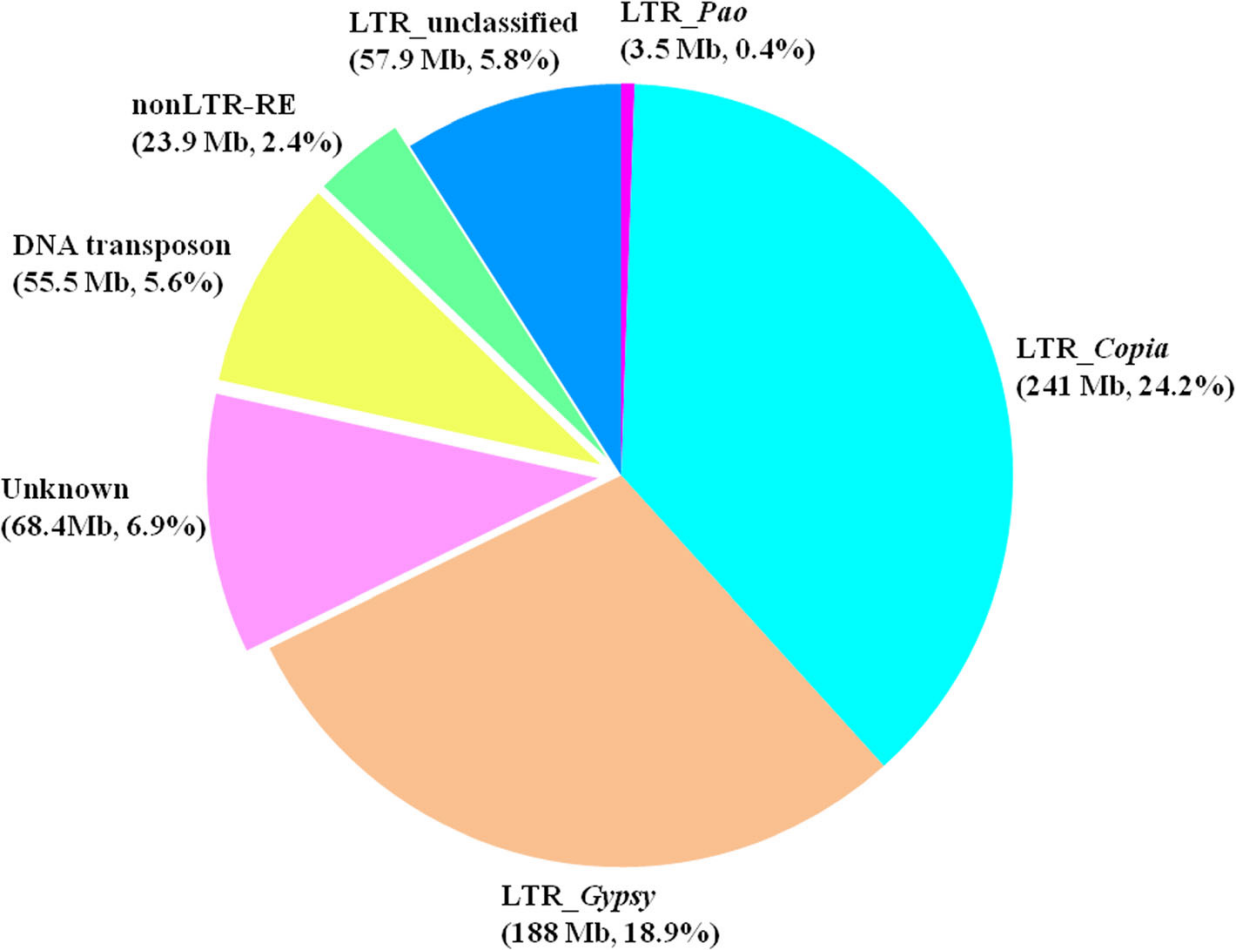
TE annotation of spinach draft genome. Whole TE fraction, total length, and genome proportion of nonLTR-RE, DNA transposon, unknown elements, and LTR-RE subdivided into each superfamily are shown

Based on combined strategies including de novo-, signature-, and homology-based methods, overall, TEs masked approximately 64.05% (637,890,846 bp) of the spinach draft genome. The LTR-REs, non-LTR-REs, and DNA transposons occupied 490, 23.9, and 55.5 Mb DNA sequences, accounting for 49.2, 2.4, and 5.6% of the genome, respectively. A total of 68.4 Mb TE sequences were unclassified, representing 6.9% of the genome (Fig. 4a). Out of the most abundant TE element in LTR-REs, *Copia* and *Gypsy* represented approximately 24.2 and 18.9% of the genome, respectively (Fig. 4b).

### Chromosome localization analysis of LTR-REs

**Fig. 5:**
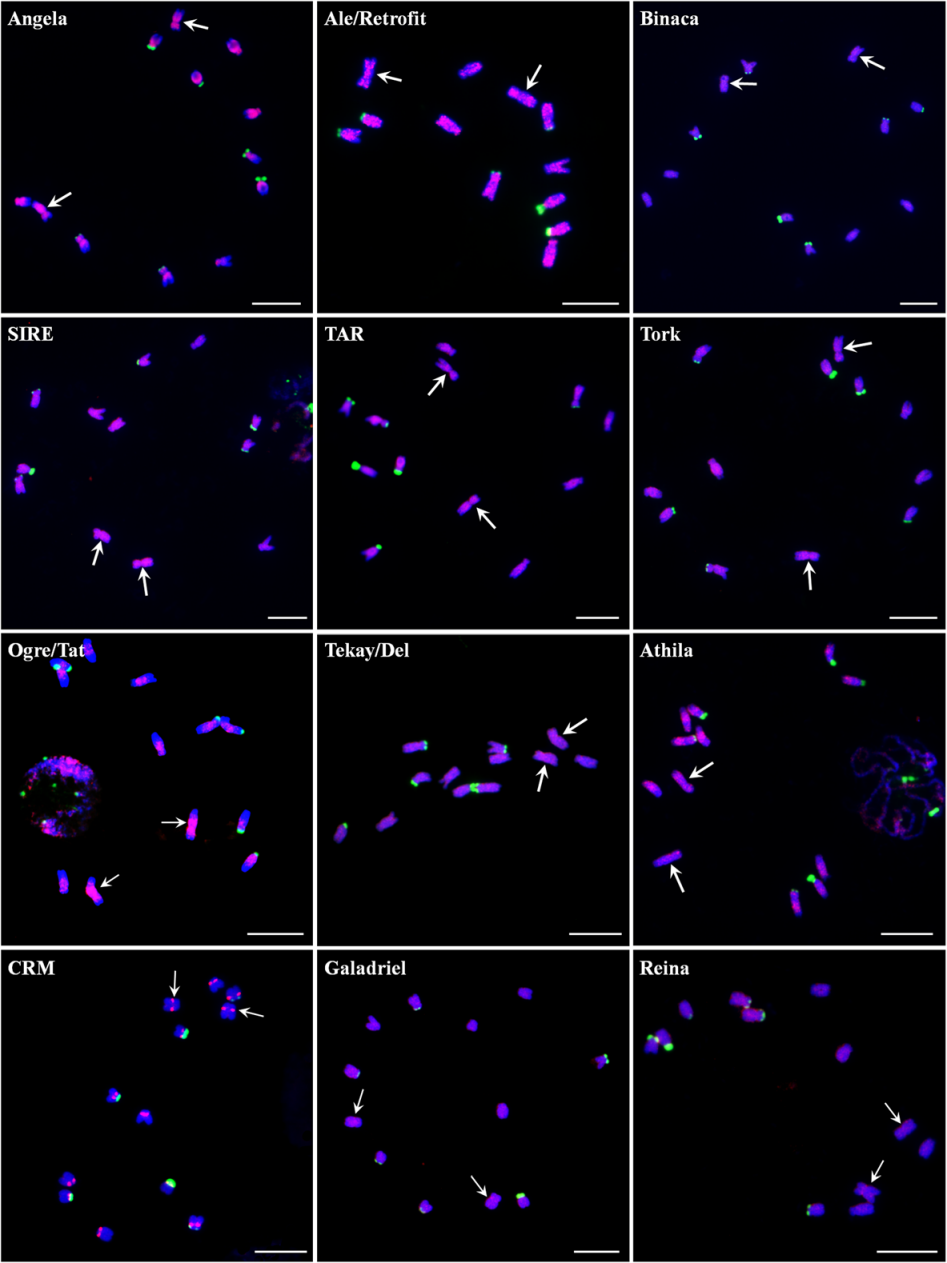
Distribution patterns of different LTR-RE lineages on spinach chromosomes. The RT sequences of each lineage were labeled with Texas red (red signal), 45S rDNA was labeled with Chroma Tide Alexa Fluor 488 (green signal), and the chromosomes were counterstained with DAPI (blue). Arrows indicate the sex chromosomes with more brighter signals using probe of Ogre/Tat lineage RT sequence. Arrows indicate the sex chromosomes. Bars = 10 μm

We analyzed the chromosome distribution patterns of all the lineages of LTR-REs on spinach mitotic chromosomes. The results showed that elements from distinct evolutionary lineages exhibit different patterns of genomic distribution. Most of the lineage-based elements were dispersed over all of the chromosomes; these included four lineages of the *Copia* superfamily (Ale/Retrofit, SIRE, TAR, and Tork) and five lineages of the *Gypsy* superfamily (Tekay/Del, Athila, Galadriel, and Reina) (Fig. 5). The other three lineages (Angela, Binaca, and Ogre/Tat) were mainly occupied the pericentromeric regions of all chromosomes, and the signals of Ogre/Tat on sex chromosomes were brighter and more intensive than on other autosomes (Fig. 5). CRM elements were predominantly located at the centromeric region of all chromosomes (Fig. 5). In addition, a probe derived from Ivana elements failed to give visible signals, most probably due to relatively low copy number.

### Satellite identification and chromosome location analysis

**Fig. 6:**
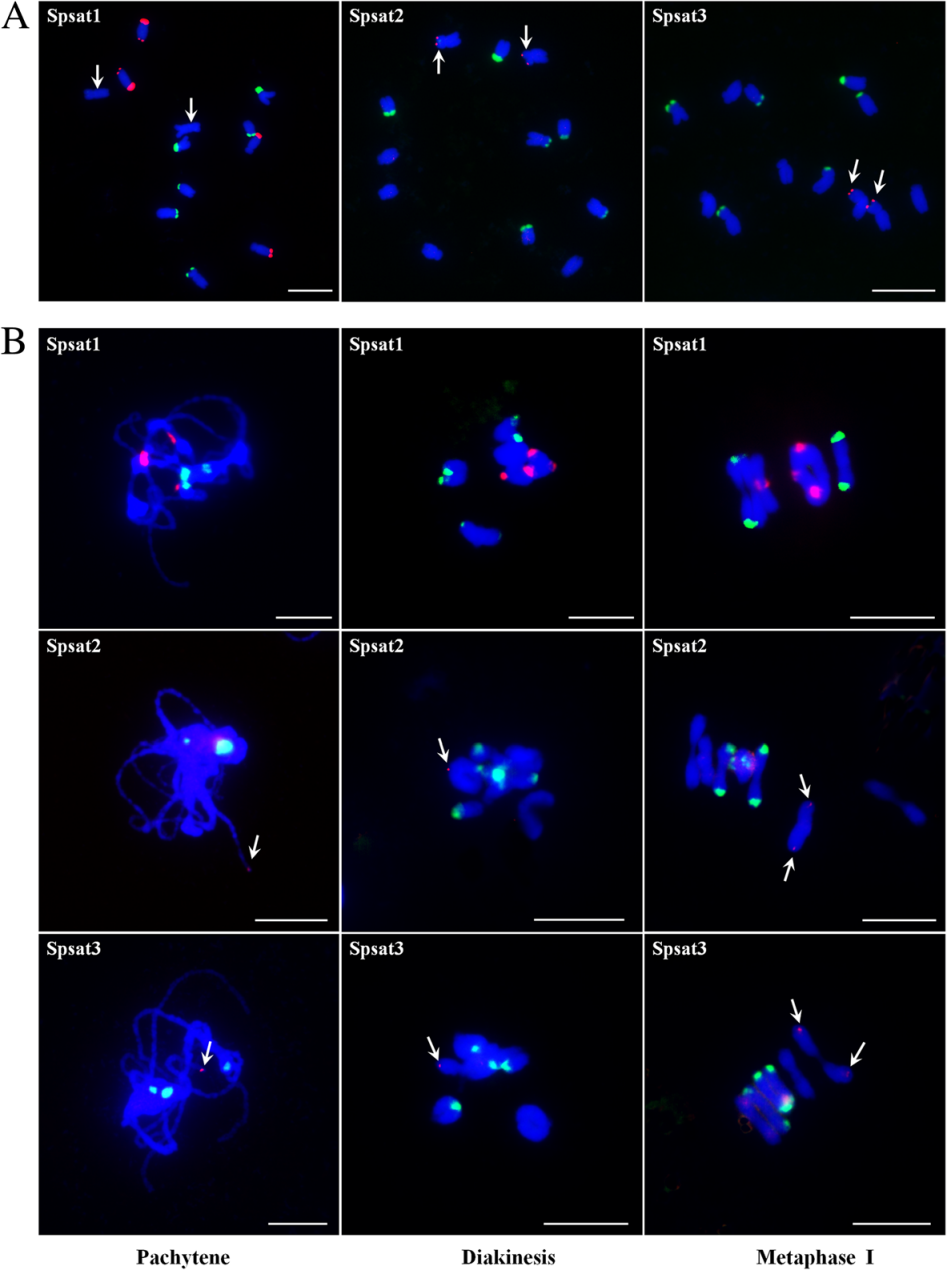
FISH mapping of three satellites on both mitotic and meiotic chromosomes in spinach. **a** FISH analysis of Spsat1, Spsat2, and Spsat3 on mitotic chromosomes. Arrows indicate the sex chromosomes (Spsat1) or the signals on the sex chromosomes (Spsat2 and Spsat3); **b** FISH analysis of Spsat1, Spsat2, and Spsat3 on meiotic chromosomes; three typical phases: pachytene, diakinesis, and metaphase I are shown. Arrows indicate the signals of the satellite DNAs. The satellite DNAs were labeled with Texas red (red signal), 45S rDNA was labeled with Chroma Tide Alexa Fluor 488 (green signal), and the chromosomes were counterstained with DAPI (blue). Bars = 10 μm

We used TAREAN for identification of satellite DNAs using unassembled reads. A set of randomly selected 2,000,000 reads with average length of 301 bp, amounting to 0.6 × spinach genome equivalent, was used for analysis. The results showed that in addition to rDNA, three clusters (CL51, CL162, and CL208) were identified as satellite DNAs. We designated them as Spsat1, Spsat2, and Spsat3, respectively. These clusters featured a star-like or circular graph topology (Additional file 2). The Spsat1 was estimated to make up 0.47% of the genome. The cluster showed ~ 52 bp monomers, and the monomers presented remarkably high similarity (Additional file 2). Searching GenBank revealed no similarities to other known sequences. FISH on mitotic chromosomes showed that the signals of Spsat1 concentrated on the telomeric regions of two pairs of chromosomes. In one pair of chromosomes, the telomeric region of the short arm showed strong signals, whereas that of the long arm showed relatively weak signals. In another pair of chromosomes, signals were distributed on the telomeric region of the long arm (Fig. 6a). FISH on meiotic chromosomes showed that three signals, one large and two small, were detected on the pachytene chromosomes. In diakinesis period, two bivalent chromosomes, one with signals at both ends and one with signals at one end, were observed. In metaphase I, the strong signals on the short arm of one pair of chromosomes were directed poleward, whereas the other signals were at the middle of the bivalent chromosomes (Fig. 6b).

The other two satellites Spsat2 and Spsat3 showed ~ 365 and ~ 325 bp monomers, respectively. Similar to Spsat1, the clusters were all unknown or spinach-specific sequences. Mitotic FISH revealed that these clusters were located at the telomeric regions of the short arm of one pair of chromosomes (Fig. 6a). According to a comparison of the chromosomes, this pair of chromosomes is the largest, that is, they are sex chromosomes. For meiotic FISH analyses, one clear signal near the end of one chromosome was detected in pachytene and diakinesis periods (Fig. 6b). In metaphase I, the signals on one pair of chromosomes were directed poleward (Fig. 6b), indicating the signals were closer to the centromeres of the chromosomes.

**Fig. 7:**
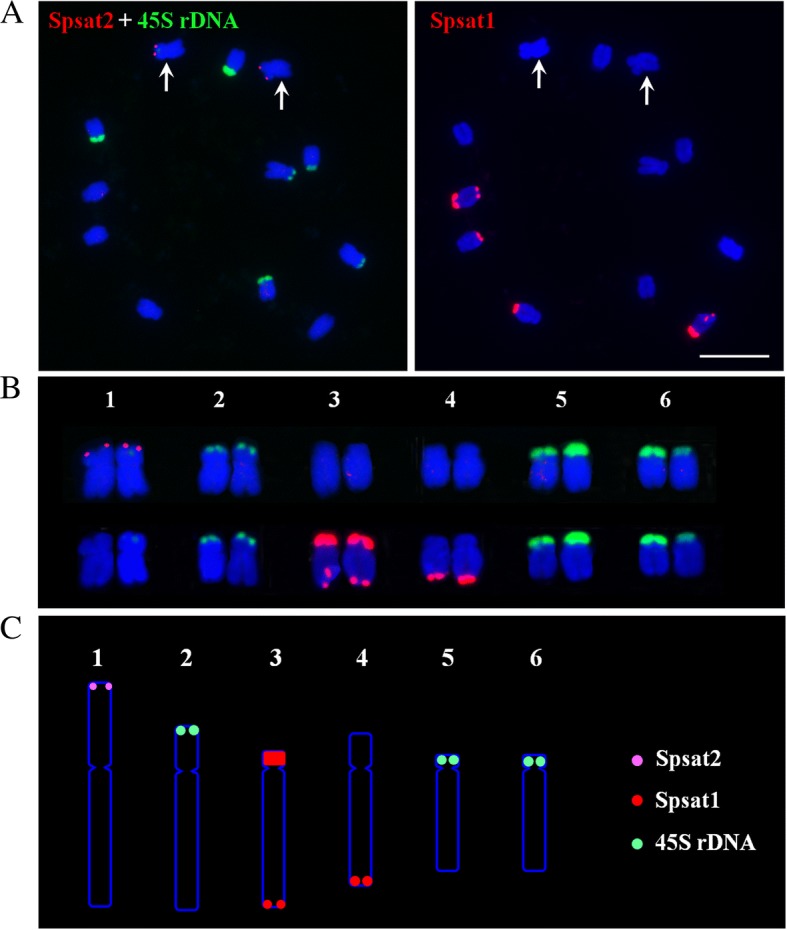
Karyotype and ideograph for spinach mitotic metaphase chromosomes. **a** FISH with Spsat2 (red) and 45S rDNA (green), and the same spread was probed with the probe of Spsat1 (red). **b** Karyotype analysis of spinach based on the size and FISH signal pattern. **c** Ideogram shows the positions of Spsat2 (purple), Spsat1 (red), and 45S rDNA (green). Bars = 10 μm

To precisely identify each pair of homologous chromosomes and obtain detailed molecular karyotype of spinach, we performed sequential FISH using satellite DNA sequences (Spsat2, Spsat1, and 45S rDNA) as probes. Based on the signals of the three probes, an accurate karyotype of spinach was established (Fig. 7). The Spsat2 signals concentrated on the telomeric regions of the short arm of chromosome 1 (sex chromosome). The Spsat1 signals were located at the telomeric regions of both the telomeric regions of chromosome 3 and the long arm of chromosome 4. The 45S rDNA was mainly distributed on the telomeric regions of the three other pairs of chromosomes, i. e. chromosomes 2, 5, and 6.

## Discussion

### TE annotation of spinach draft genome

Using combined annotation strategies, TEs represent 64.05% of the spinach draft genome, and this value is slightly lower than that of a previous report on the same genome [14]. Compared with that previous study, we used more TE detection software with more stringent analyses and could thus obtain more reliable results. The proportion of TE fraction of different plant genomes presented significant variation. For example, TEs represent less than 20% of the *Arabidopsis* genome [21] but occupy more than 85% of the wheat genome [18]. In general, the quantity of TEs, especially LTR-REs, correlates well with the genome size of higher plants [7]. Spinach possesses a medium-large genome with 996 Mb, and the TE fraction proportion is generally in line with the trend. Among the TEs, REs are usually far more abundant than transposons, whereas LTR-REs are predominant TE elements in plant genomes [7]. As expected, REs represented 49.2% of the spinach genome, whereas DNA transposons occupied a small proportion (5.6%). The prevalence of REs, particularly LTR-REs, was mainly due to their intrinsic proliferation characteristics [22]. Among LTR-REs, *Copia* elements were more abundant than *Gypsy* elements in the spinach genome. This result disagrees with previous findings, which showed the prevalence of *Gypsy* elements in spinach genome [15]. This result may be caused by the different annotation methods used. A large difference in the proportion of *Copia* and *Gypsy* elements was observed among different plant genomes. Different ratios between *Gypsy* and *Copia* element frequencies were reported, with values ranging from 10:1 in *Gossypium arboreum* [23] to 1:4 in *Elaeis guineensis* [24]. Among the 86 plant genomes we collected in a previous paper [7], *Gypsy* elements predominate in 64 genomes, whereas the other 22 are *Copia*-biased. This observation indicates that different genomes show unique retroelement expansion patterns, which are mainly due to different evolutionary processes within each plant species [25].

### Diversity and dynamics of full-length LTR-retroelements in the spinach genome

Given that LTR-retroelements are usually far more abundant than other types of TEs, and identification of full-length elements benefits the investigation of structural variability, diversity, and phylogenetic evolution of TEs in spinach genome, we analyzed full-length LTR-REs in detail. In this study, the most abundant full-length LTR-REs belonged to *Copia*, followed by *Gypsy*, and the remainder elements were unknown. Among the lineages, Angela and Ale *Copia* REs were more redundant than the other *Copia* lineages, whereas Ogre/Tat was the most redundant *Gypsy* lineage. These results suggest that the proliferation of certain lineages, such as Angela and Ogre/Tat, contributes significantly to spinach genome evolution. The prevalence of particular repeat lineages or families differs dramatically among different plant species. In numerous cases, a limited number of repetitive types are highly amplified. For example, five families of LTR-REs represent approximately 80% of the maize RE repertoire [1, 26], and a single Ty3-*Gypsy*-like RE accounts for approximately 38% of the genome of *Vicia pannonica* [27]. The mechanisms behind the proliferation of several RE families or lineages are poorly understood, and the most accepted explanation is that these families or lineages lost their silencing cellular mechanisms of the host genome [28, 29].

The LTR-REs showed considerable structural diversity. During retrotransposition, the active LTR-REs should exhibit all the elements that facilitate retrotransposition, including those of terminal LTRs, PBS and PPT sites, and all the necessary protein domains. However, as selective pressure usually features no or slight influence on TEs, TEs usually undergo rapid evolution process, such as truncations, nested insertions, and mutations [30]. These variationss result in the structural diversity of TEs. We discovered that only 145 full-length REs possessed all the required elements for retrotransposition, indicating that most of the REs in spinach are inactive. The inactivation of TEs is a protection mechanism to genome stability because a high level of TE activity is widely believed to induce genome instability, which is harmful for the genome [31].

The two LTRs of a retroelement were identical at the time of insertion and subsequently diverge due to random mutations, thus facilitating the estimation of insertion time of REs [26]. The mean RE insertion date was 1.42 MY, and the majority of retrotransposons were accumulated within the last two million years, indicating the very recent and probably still occurring RE burst. The recent RE proliferation events might exhibit important influence on spinach genome structure and evolution. A similar time course of RE amplification wave was reported in other herbaceous species, such as rice, wheat, *Solanum lycopersicum*, and *Arabidopsis* [25, 32, 33]. In spinach, the mean insertion data of *Copia* full-length REs was lower than that of *Gypsy* REs. However, the insertion date profiles indicate that *Copia* and *Gypsy* REs have experienced similar time courses.

### Repetitive sequences and sex chromosome evolution of spinach

FISH analysis showed that Ogre/Tat lineage accumulated more in the sex chromosomes than in autosomes. In addition, two satellite DNAs were exclusively located in sex chromosomes. These results suggest that repetitive sequences, including TEs and satellites, accumulated more in sex chromosomes than in autosomes. Several studies have demonstrated that repetitive sequences accumulate in sex chromosomes in both plants and animals [10, 34]. For example, in papaya, repetitive sequences occupy 79.2% of MSY and 67.2% of the X chromosome counterpart, whereas the ratio of repetitive sequences in the entire genome is 51% [35, 36]. In another dioecious plant *Rumex acetosa*, a number of satellites are located in sex chromosomes or Y chromosomes only [37]. The TEs and TE-derived repetitive sequences are believed to participate in nearly all the main evolutionary steps of sex chromosome evolution, such as recombination suppression, heterochromatization, chromosome morphology and structure alteration, and Y chromosome degeneration [10]. In addition, TEs and related repetitive sequences may regulate sex determination and differentiation of plants. For example, in the monoecious plant melon, one TE is inserted into transcription factor *CmWIP1*, leading to the methylation of the flanking transcription factor sequence of the TE and causing the development of unisexual male flowers [8]. Spinach possesses a pair of young homomorphic sex chromosomes (X and Y). The sex chromosome-biased TEs and sex chromosome-specific satellites may play a role in sex chromosome formation and evolution in spinach.

By contrast, it should be noted that several repetitive seqeunces are ubiquitously distributed in autosomes but are absent in sex chromosomes [38–40]. For example, one family of Ogre/Tat lineage is nearly absent in the Y chromosome but is distributed widely in autosomes and X chromosome in *Silene latifolia* [39]. We also found that a satellite DNA is distributed at the telomeric regions of autosomes and X chromosome, but not in Y chromosome in *Humulus scandens* (our unpublished results). Thus, the relationship between repetitive sequences and the evolution of plant sex chromosomes is complicated. Based on the current limited reports [10, 38–40], it seems that the accumulation or depletion of which type of repetitive sequences is species-specific, which is consistent with the fact that sex chromosomes have evolved many times indepedently in plants [41].

**Fig. 8:**
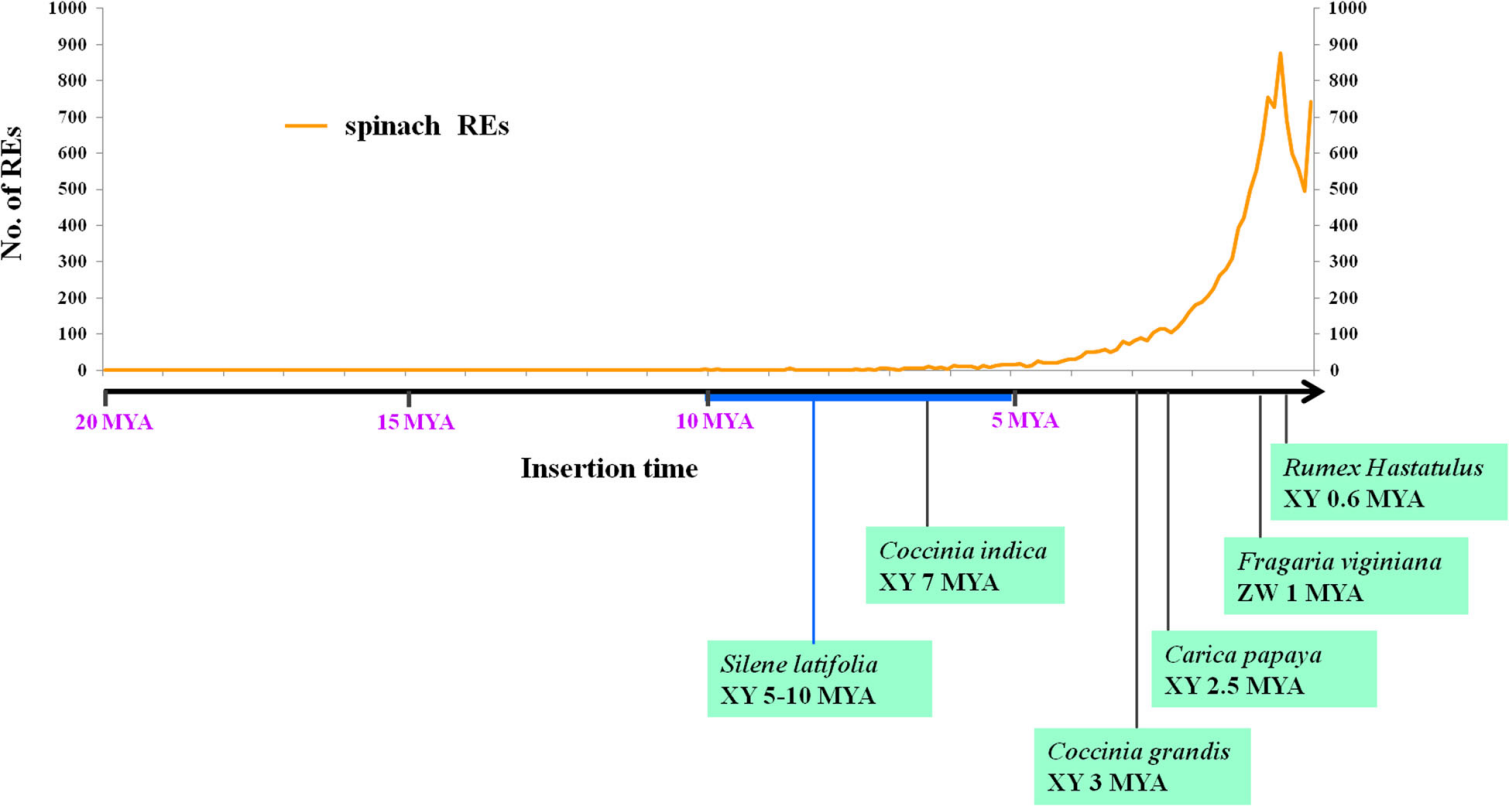
Evolutionary age of the reported sex chromosomes of dioecious plants and insertion age of LTR-REs in spinach

Sex chromosomes in most dioecious plants evolved much more recently compared with most of animal sex chromosomes. The evolution time of human sex chromosome is approximately 240–300 MY, whereas most of the plant sex chromosomes emerged within the past 25 MY [42, 43]. For instance, sex chromosomes of *Silene latifolia* and *Coccinia indica* evolved less than 10 MY [44, 45]. The homomorphic sex chromosomes of *Carica papaya*, *Fragaria viginiana*, and *Rumex hastatulus* evolved even more recently, with evolution times of 2.5, 1, and 0.6 MY, respectively [46–48] (Fig. 8). Presently, no study reported the evolution time of sex chromosomes in spinach. However, given that X and Y chromosomes are homomorphic, and the YY individual can survive, we posit that the sex chromosomes of spinach must be young. Studies have reported that plant REs mainly evolved within the last 1–12 MY [49, 50]. Therefore, the majority of the sex chromosomes of dioecious plants emerged after plant RE evolution. Thus, we propose that sex chromosome origin and evolution may be closely related to the burst of repetitive sequences, mainly REs. In this study, the rapid amplification of REs occurred within the last two million years, and this RE proliferation event may be involved in the origin and evolution of spinach sex chromosomes. In the future, with the complete assembly of sex chromosomes, we can investigate the relationship of repetitive sequences and sex chromosome formation and evolution in detail.

### Molecular karyotypic analyses of spinach

Although spinach is a popular vegetable and a model dioecious plant for studying sex chromosome evolution and sex determination mechanism, few cytogenetic studies were carried out, and detailed molecular karyotype analyses that reliably distinguish each chromosome of spinach have not been conducted. Chromosome identification is essential for cytological analyses and subsequent studies in genomics, taxonomy, and evolution of sex chromosomes, establishing a bridge between visible landmarks and genetic or physical map features. The satellites identified in this study provide good markers for karyotyping analysis. Based on satellite identification and FISH analysis, we obtained three useful satellite DNAs (Spsat1, Spsat2, and Spsat3) which can be used as cytogenetic markers. Together with rDNAs, one or two signals were detected on each chromosome. We can now easily identify all six somatic metaphase chromosomes by the position of FISH signals. Based on sequential FISH, an integrated metaphase chromosome karyotype was established, providing a valuable basis for future cytogenetic and genomic studies.

## Conclusions

In conclusion, this study presents a comprehensive description of the repetitive sequence organization of spinach, an important vegetable and dioecious plant species. We reliably annotated the TE portion of the spinach draft genome based on combined strategies, with LTR-REs representing 49.2% of the spinach genome. The full-length LTR-RE elements allowed us to investigate the structural variation, phylogenetic relationship, and insertion dynamics of the most abundant genome fraction. Our TE database will serve as resource in future studies aimed at assessing the possible contribution of TEs to genome structure and evolution. FISH analysis showed that one lineage of LTR-RE and two satellites accumulated on sex chromosomes, suggesting that repetitive sequences may play important roles in spinach sex chromosome evolution. The satellites identified in this study provide good markers for future cytogenetic analysis of spinach.

## Materials and methods

### Plant materials

The spinach variety Daye (II 9A0002) was used in this study. Seeds were sown and grown in a garden field of Henan Normal University under natural conditions. Total genomic DNA was extracted from young leaves using traditional cetyl trimethylammonium bromide method.

### Identification of TEs

The spinach draft genome was downloaded from http://www.spinachbase.org/?q=download. We used LTRharvest [51] to identify LTR-REs, and the parameters included the following: LTR size of 100–5000 bp, minimum distance between LTRs of 1000 bp, maximum distance between LTRs of 20,000 bp, flanking by dinucleotides TG and CA at 5′ and 3′ of each LTR, similarity of 60%, TSD of 4–8 bp, and other default parameters. The internal features of the identified putative LTR retroelements were annotated by using LTRdigest program [52]. A database of tRNAs using tRNAscan-SE (version 1.3.1) [53] was constructed to predict the location of PBS, and the hidden Markov model profiles were downloaded from the *Gypsy* [54] (http://gydb.org/index.php/Main_Page) and Pfam databases [55] (http://pfam.xfam.org 31.0). The putative full-length LTR-REs with typical LTR-RE features, that is, with a putative 15–18 nt PBS or a 10–30 nt PPT upstream of the 5′ end of the 3′ LTR or possessing one of the typical RE protein domains (GAG, protease, RT, RH, and INT), were filtered for further analysis; the remaining LTR-REs were discarded. The identified LTR-REs were first classified into *Copia* or *Gypsy* superfamilies according to the order of the RT and INT domains, that is, the *Copia* candidates showed the INT-RT order, whereas the *Gypsy* ones showed the RT-INT order. The protein domains of the identified full-length LTR-REs belonging to *Gypsy* or *Copia* superfamiles were then extracted using RepeatExplorer [56] and were used as reference datasets for further BLASTX queries to classify previously unclassified elements. Finally, the still unclassified elements were further classified by BLASTN and BLASTX searches against Viridiplantae TE database retrieved from Repbase (http://www.girinst.org/repbase, 23.07) and public sequence databases (non-redundant nucleotide and protein National Center for Biotechnology Information (NCBI) databases). We used E value thresholds of E < 10^− 10^ and E < 10^− 15^ for BLASTN and BLASTX, respectively.

For non-LTR-REs, MGEScan-nonLTR program [57] was used with default parameters. The results of this program were well-classified superfamilies of non-LTR-REs. To detect Helitron DNA transposons, HelitronScanner software [58] was used. We used the parameters of headscore 8 and tailscore 10 to obtain reliable results. Small non-autonomous DNA transposon elements were identified by using MUSTv2 program [59] and MITE_Hunter [60]. The results obtained by these software were combined and annotated by using RepeatClassifier program (v1.0.10), which is a part of the RepeatModeler package, and then finally checked manually.

### Phylogeny and classification of LTR-REs

The LTR-REs were classified into known lineages and clades according to the phylogenetic relationships of their RT protein domains, which were extracted from full-length REs using the RepeatExplorer platform. After removing redundant sequences using CD-hit [61], multiple alignment of the consensus RT domain sequences was carried out using MUSCLE [62]. The alignment was used to construct phylogenetic trees by using FastTree [63]. The trees were drawn and further edited using FigTree software.

### Insertion time estimation of LTR-REs

The 5′- and 3′-LTRs of each putative full-length LTR-REs were compared to estimate the insertion age of LTR-REs [64]. After the two LTRs of each RE were aligned by using program “Stretcher” (EMBOSS package) [65], the nucleotide distance between two LTRs were measured using the Kimura two-parameter method [66]. An average substitution rate (r) of 1.3 × 10^− 8^ substitutions per synonymous site per year and insertion time (T) formula T = k/2r were then employed to measure insertion time [32].

### TE annotation of the spinach genome

TE annotation was performed by using RepeatMasker based on libraries generated by different strategies: de novo-based, signature-based, and homology-based methods. De novo identification of TEs was performed using RepeatModeler (http://www.repeatmasker.org/RepeatModeler, version 1.0.10). The consensus families generated by RepeatModeler (Additional file 3) were used as a custom library to mask the spinach genome by RepeatMasker (v4.0.7). The spinach genome was then masked using the library of previously classified TE sequences identified by signature-based methods (Additional file 4). The unmasked sequences were further analyzed by RepeatMasker using the filtered consensus family sequences as a custom library. Finally, the consensus TE sequences from Repbase were used as a library to mask the remaining unmasked sequences using RepeatMasker. The results of the above three steps were combined and analyzed.

### Identification of satellite DNAs

A set of whole genome Illumina Miseq paired-end reads with average length of 301 bp was downloaded from NCBI with Sequence Read Archive accession number of SRR4447192 [67]. After filtering using HTQC (v1.92.1) [68], a randomly selected dataset containing 2,000,000 reads, which represented 0.6× spinach genome, was used for graph-based clustering analysis using TAREAN online pipeline [69]. Clustering analysis was performed using a threshold of 90% similarity over at least 55% of the sequence length. Clusters containing satellite repeats were identified based on graph topology and software estimation results. The genome proportion of each putative satellite DNA cluster was calculated as the percentage of reads, which is the number of reads in each cluster divided by all the reads used in the graph-based clustering. The logo for the satellite sequence was generated by Web-logo [70].

### Preparation of probes for FISH

The FISH probes used in this study were produced from two sets of data, that is, the RT domains of the lineages of LTR-REs and satellites. To investigate the distribution of major lineages of LTR-REs, we amplified RT domains of *Copia* and *Gypsy* REs using designed specific primer sets (Additional file 5). PCR products were checked by gel electrophoresis, and the desired bands were cleaved, cloned into pEASY-T1 vector (Transgene, Beijing, China), and transformed into competent *Escherichia coli* cells. The positive clones were screened and sequenced (Sangon Biotech, Shanghai, China) to verify the presence of specific RT domains in the clones. Clones with at least 90% similarity to the corresponding reconstructed contigs were PCR-amplified and labeled with Texas-red-dCTP (PerkinElmer, Waltham, Massachusetts, USA) using nick translation method as described previously [71]. The monomers of satellites were synthesized with 5′-Texas-red modification (Invitrogen, Shanghai, China). For improved characterization of the chromosomes, 45S rDNA was labeled with Chroma Tide Alexa Fluor 488–5-dUTP (Invitrogen) for FISH.

### Chromosome preparation and FISH analysis

Mitotic metaphase spreads were prepared from meristem root tip cells following previously used procedures [13] with minor modifications. Briefly, spinach seeds were cultured on moist papers in dishes at 25 °C. After 1–2 days, root tips with approximately 1 cm length were cut and pretreated with nitrous oxide gas for 2 h. The root tips were then fixed in ice-cold 90% acetic acid for 10 min and stored in 70% ethanol at − 20 °C. The root tips were washed in ice-cold 1 × citric buffer for 10 min. The root sections with actively dividing region were excised and incubated in an enzyme mixture containing 1% pectolyase Y 23 (Yakult Pharmaceutical, Tokyo, Japan) and 2% cellulose Onozula R10 (Yakult Pharmaceutical) for 2 h at 37 °C. After digestion, the root sections were washed in ice-cold TE and 100% ethanol twice in sequence. The root sections were fine-broken with a needle and vortexed at 4000 rpm for 20 s. The cells were collected by centrifugation and resuspended in 100% acetic acid to prepare a cell suspension. The cell suspension was dropped onto glass slides in a moist box and dried. The slides were checked under a phase-contrast microscope. For meiotic spread preparation, immature flower buds measuring approximately 0.5 mm in length were directly fixed in ethanol/acetic acid (3:1) for 24 h and stored in 70% ethanol. The anthers were isolated and used for spread preparation. The procedure was the same as that for mitotic spread preparation.

The selected slides with well-spread metaphase chromosomes or desired stage of mitotic process were UV-crosslinked for 2 min. A probe solution containing in 2 × SSC and 1 × TE was then added on the slides. After denaturation in boiling water for 5 min, the slides with probe were incubated at 55 °C in a humid chamber for 8–12 h. The slides were then washed thrice in 2 × SSC, with each washing lasting for 5 min, at 50 °C and finally counterstained with 4′,6-diamidino-2-phenylindole (DAPI) (Vector Laboratories, Burlingame, USA). The FISH images were captured with an ANDOR CCD under an Olympus BX63 fluorescence microscope. The images were processed by Adobe Photoshop 7.0.

## Additional files

Additional file 1: Annotation of full-length LTR-REs in spinach genome. (XLS 3011 kb)

Additional file 2: Topological layout and consensus sequence of satellite DNAs. (DOC 1441 kb)

Additional file 3: TE concensus sequences generated by RepeatModeler in spinach genome. (FASTA 1073 kb)

Additional file 4: TE dataset detected by signature-based methods in spinach genome. (FASTA 122852 kb)

Additional file 5: The primers used for amplification of the RT sequences of lineages in spinach. (DOC 42 kb)

## Abbreviations

FISH: Fluorescence in situ hybridization
INT: Integrase
LTR: Long terminal repeat
MITE: Miniature inverted repeat transposable element
PBS: Primer-binding site
PPT: Polypurine tract
RE: Retrotransposon
RH: RNAseH
RT: Reverse transcriptase
TE: Transposable element
TIR: Terminal inverted repeat
TSD: Target site duplication
