# Supplementary material for: The landscape of transposable elements and satellite DNAs in the genome of a dioecious plant spinach (Spinacia oleracea L.)
Source: Mob DNA. 2019 Jan 18;10:3. doi: 10.1186/s13100-019-0147-6 (PMC6337768; doi:10.1186/s13100-019-0147-6)
Supplement: Supplementary file 2 — Topological layout and consensus sequence of satellite DNAs. (DOC 1441 kb) [file 13100_2019_147_MOESM2_ESM.doc]

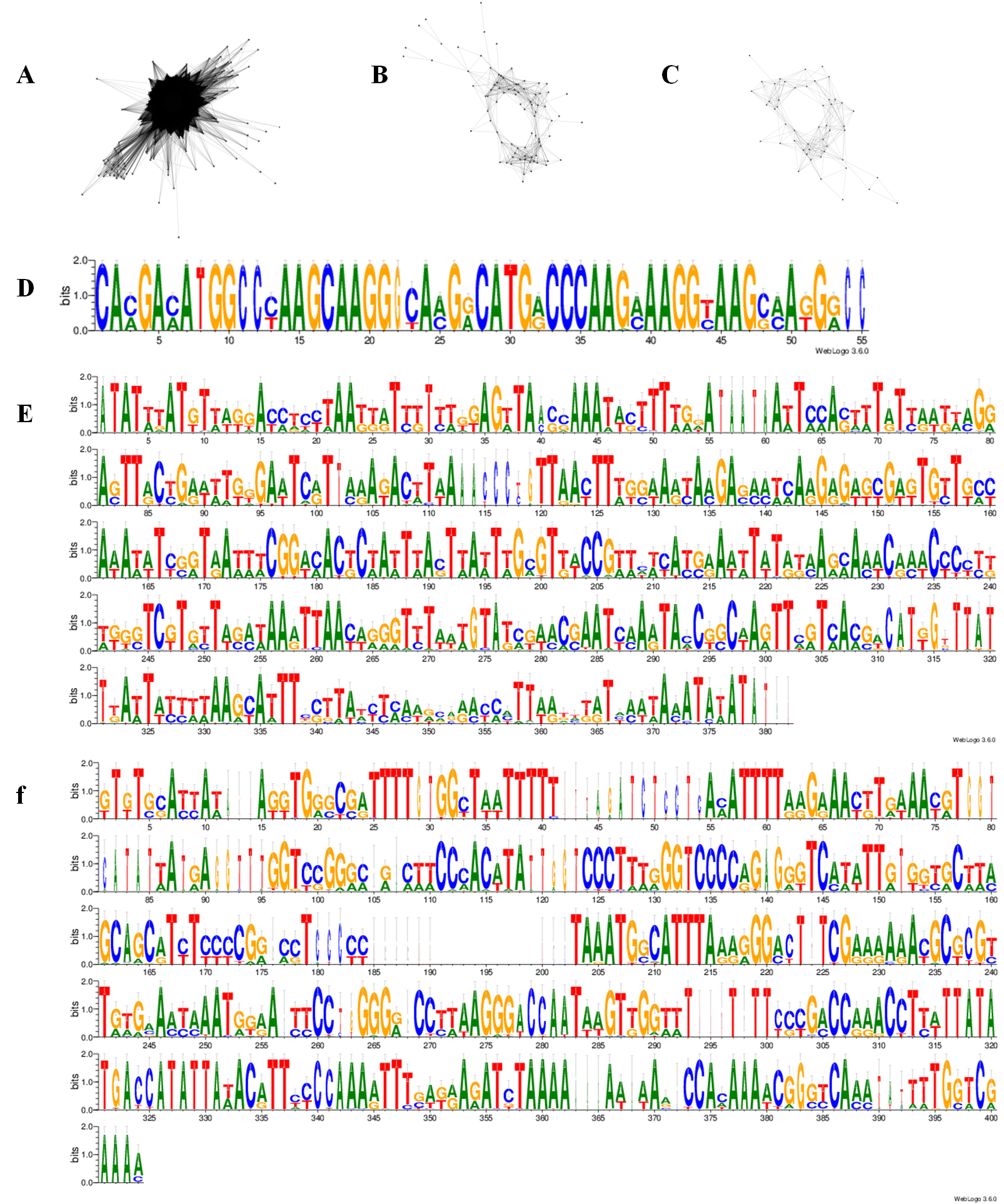


**Figure S1. Topological layout and consensus sequence of satellite DNAs.**

A, B and C indicate topology layouts of Spsat1, Spsat2, and Spsat3, respectively. D, E. and F show consensus seqeunces of Spsat1, Spsat2, and Spsat3, respectively.
