# Supplementary material for: The landscape of transposable elements and satellite DNAs in the genome of a dioecious plant spinach (Spinacia oleracea L.)
Source: Mob DNA. 2019 Jan 18;10:3. doi: 10.1186/s13100-019-0147-6 (PMC6337768; doi:10.1186/s13100-019-0147-6)
Supplement: Supplementary file 5 — The primers used for amplification of the RT sequences of lineages in spinach. (DOC 42 kb) [file 13100_2019_147_MOESM5_ESM.doc]

Table S2 The primers used for amplification of the RT sequences of lineages in spinach

| Name | Primer sequence (5′-3′) |
| --- | --- |
| Angela | F: CACTCATTTGTCTACGCTC |
|  | R: CAACCGGAAAGGTACTTAGG |
| Ale/Retrofit | F: AATGCTGCTTCCCAATGCTC |
|  | R: CCTCAAATACTCCCTATCCTATC |
| Binaca | F: AGGTCTCGTATGACTAGCAAG |
|  | R: ATGGAAGAAAGCAATTGAGG |
| SIRE | F: CCTGCACTCATCTCAGCTTG |
|  | R: CTCTCCAATGGATCTGATCG |
| Ivana | F: CGAGCTAGAATCCATCATGAAG |
|  | R: TGCTTGAGTGCTCGTCTATG |
| TAR | F: GATTGACAGACAGGATCACC |
|  | R: TATAAGGCCAGGATTGTGGTAA |
| Tork | F: TTGTTGACAGATGCCGGTGA |
|  | R: GACAATCCTCCTTAGTGAGC |
| Ogre/Tat | F: GAGAGGACGGGTGGCTTG |
|  | R: CGCTAGATCCCAAAATGACG |
| Tekay/Del | F: TGATCTTAGCAGGGTCAACT |
|  | R: TACATTAGGCCAAGTGCATC |
| Athila | F: ATTAGGCCACGATTTGATAG |
|  | R: TGCTTGATGCGGGTATTGTG |
| CRM | F: ACTTCCTTTGACTCCATGC |
|  | R: GCTGCCTATCGTTGTAATCC |
| Galadriel | F: TTGCCAATCACGTATAGCTT |
|  | R: AGATGATTTGCTGAGAGGTG |
| Reina | F: CTCCTTGACTATAGTGGGAAC |
|  | R: GGAACCTAACTCAAGACCTG |
